# Symptoms Associated With Detection of Viral Versus Bacterial Pathogens in Outpatients With Lower Respiratory Infections

**DOI:** 10.1111/irv.70229

**Published:** 2026-03-15

**Authors:** Mark Ebell, Dan J. Merenstein, Bruce Barrett, Cassie Hulme, Sarah Walters, Caroline Hamer, Michelle Buhr

**Affiliations:** ^1^ Department of Family Medicine, College of Human Medicine Michigan State University East Lansing Michigan USA; ^2^ Department of Family Medicine Georgetown University Washington DC USA; ^3^ Department of Family Medicine and Community Health University of Wisconsin Madison Wisconsin USA; ^4^ Division of Pediatric Urology Cincinnati Children's Hospital Cincinnati Ohio USA; ^5^ Department of Gynecology and Obstetrics, School of Medicine Emory University Atlanta Georgia USA

**Keywords:** acute bronchitis, antibiotic stewardship, pneumonia, respiratory tract infection

## Abstract

**Purpose:**

This study aims to identify symptoms that predict a high likelihood of viral infection, so these patients could be triaged for home care to avoid antibiotics.

**Methods:**

We recruited adults presenting to US primary or urgent care sites with a chief complaint of cough and symptoms consistent with LRTI. Data collected included demographics, comorbidities, symptoms, and 46 viral and bacterial respiratory pathogens by PCR. Chi‐square tests were done to evaluate the association between individual symptoms and viral versus bacterial infections. Symptoms with a *p* < 0.10 for the association were retained for logistic regression. We created four regression models using stepwise backward elimination at *p* < 0.20, with viral infection, bacterial infection, and mixed infection as the dependent variables. A simple risk score was created assigning positive and negative points for viral and bacterial symptoms.

**Results:**

We enrolled 718 adults with acute cough and obtained valid PCR specimens for 618. Four symptoms were significantly more likely with viral infections and less common in bacterial: coryza, confusion, fever, and chest congestion. Three symptoms were more likely with bacterial infections and less likely with viral infections: presence of sputum, sputum that is colored, and double‐sickening (feeling better but then worsening). A simple risk score identified patients with a low (29%), moderate (56%), or high (79%) likelihood of having only viral pathogens detected.

**Conclusions:**

Seven symptoms were identified that could help primary care clinicians distinguish between viral and bacterial LRTI. A simple risk score is proposed but requires prospective validation.

## 1 Introduction

While many patients with a cough do not seek medical attention, many others seek care at primary care clinics, urgent care centers, and emergency departments. Patients with viral lower respiratory tract infections (LRTIs) do not benefit from antibiotics. While specific antiviral treatments exist for influenza and COVID‐19, they are of limited benefit or are not recommended for average‐risk patients, and in any case, home tests are available for those infections [1]. Therefore, identifying patients more likely to have a viral pathogen causing their symptoms could be one way to triage patients and encourage them to initially try self‐care at home.

Previous studies have identified colored sputum [2], fever, headache, diarrhea, rhinitis [3], and focal chest signs [4] as independent predictors of bacterial infection. One study found no association between reported symptoms and bacterial detection in persons with LRTI [5]. However, it would be helpful to identify symptoms of viral versus bacterial infection as a way to identify patients who can safely be managed at home in the absence of alarm symptoms.

The Enhancing Antibiotic Stewardship in Primary Care (EAST‐PC) study was a 5‐year study of patients with acute LRTI presenting in outpatient and primary care settings from 2018 to 2023 [6]. Patient‐reported symptoms, physician‐collected signs, and PCR for 46 pathogens were obtained on every patient. This paper assesses whether symptom data may be able to distinguish viral from bacterial infection, with the goal of identifying patients unlikely to benefit from a physician visit.

## 2 Methods

EAST‐PC was a prospective observational study of adult outpatients with acute cough. It was approved by the Western Institutional Review Board (IRB, #1253415) and the IRBs of each participating institution. All participants signed an informed consent form. The study was sponsored by the Agency for Healthcare Research and Quality (#1R01HS025584‐01A1). The design of the Enhancing Antibiotic Stewardship in Primary Care study has been described in detail previously and is summarized below.

### 2.1 Participants

Participants aged 18 to 75 years were identified at primary and urgent care clinics in Athens, Georgia, Washington, DC, and Madison, Wisconsin, in the United States. All study participants reported a cough for up to 14 days plus at least one of the following: shortness of breath, sputum, muscle aches, chest discomfort or congestion, measured fever or feverishness, chills, or sweats. Patients who had taken an antibiotic, antiviral, or corticosteroid in the previous 28 days, those with serious immunodeficiency, currently receiving cancer chemotherapy, or taking systemic steroids or other immunosuppressive drugs were excluded from the study. Patients with chronic lung disease other than mild to moderate asthma were also excluded. Data were collected between June 2019 and April 2023. Data collection was interrupted during the COVID‐19 pandemic and resumed on a limited basis in October 2020, with full scale data collection resuming in January 2022.

### 2.2 Baseline Data Collection

We recorded information about patient demographics, respiratory symptoms, medications, and comorbidities. Symptoms were considered present if they were rated by the patient as moderate to severe (as opposed to none or mild). Severity at baseline was evaluated using the five‐item validated Bronchitis Severity Score, which has a range from 0 to 15 points, with higher scores indicating worse severity of disease [7]. A 28‐day symptom diary and regular text messages were used to gather follow‐up data including any additional visits for the same symptoms to an outpatient clinic or emergency department.

Mid‐turbinate or anterior nares were swabbed, and the samples were processed by the Pneumonia Response and Surveillance Laboratory at the Centers for Disease Control and Prevention. A complete list of 23 viruses and 23 bacteria is shown in Appendix S1. When detected, we assumed that *Staphylococcus aureus* was a commensal organism in otherwise healthy persons [8].

### 2.3 Analysis

Chi‐square tests were done to evaluate the association between individual symptoms and viral, bacterial, and mixed infections. Symptoms with a *p* < 0.10 for the association were retained for logistic regression. We created 3 regression models using stepwise backward elimination at *p* < 0.20, with viral infection, bacterial infection, and mixed infection as the dependent variables. A simple risk score was then developed based on the regression results. All analyses were performed using Stata version 18.0 (Statacorp, College Station, TX).

## 3 Results

### 3.1 Participants

We enrolled 718 adults with acute cough and obtained valid PCR specimens for 618. Characteristics of participants are shown in Table 1. The mean age was 39 years and 66% were female. At the index visit, 179 of 618 (29%) were prescribed an antibiotic, most commonly amoxicillin/clavulanate, azithromycin, doxycycline, and amoxicillin. At baseline, 462 of 618 participants (75%) had a moderately bad or worse cough, 453 (73%) felt at least moderately unwell, 424 (69%) had at least moderate fatigue, and 333 (53.9%) had coryza.

**TABLE 1:** Characteristics of 618 patients with lower respiratory tract infection and a valid PCR specimen.

Patient characteristic	
Overall population size	618
Patient age (mean, range)	39.2, 18 to 74
Duration of cough prior to visit in days (median, IQR)	4 (3 to 7)
Female sex	408 (66.0%)
Race^a^	
White	483 (78.2%)
Asian/Pacific Islander	67 (10.8%)
Black	49 (7.9%)
American Indian/Alaska Native	7 (1.1%)
Did not respond or missing	28 (4.4%)
Antibiotic prescribed	207 (28.8%)
Amoxicillin‐clavulanate	63 (10.2%)
Azithromycin	62 (10.0%)
Doxycycline	35 (5.7%)
Amoxicillin	15 (2.4%)
Other	32 (5.2%)
Antiviral prescribed	51 (7.1%)
Oseltamivir	30 (4.9%)
Nirmatrelvir‐ritonavir (Paxlovid)	21 (3.4%)

^a^ Self‐identified, may choose more than one group.

### 3.2 Duration and Severity of Infections

Table 2 summarizes the duration and baseline severity of different types of infection. The mean baseline severity assessed using the Bronchitis Severity Score was 7.1 points out of 15 possible (95% confidence interval [CI] 6.9–7.3) and did not differ by viral, bacterial, mixed infection, or no detection. Viral infections were significantly shorter than other types of infection (14.7 days versus 16.9 for mixed, 17.3 for bacterial, and 18.4 for no detection).

**TABLE 2:** Summary data regarding type of pathogens detected, baseline severity and duration of cough.

Type of detection	Bronchitis Severity Score at baseline (range 0–15), *n* = 618		Total duration of cough (days), *n* = 401	
	Score (95% CI)	#	Mean (95% CI)	90th %ile^a^
1+ viruses, no bacteria	7.0 (6.6–7.4)	211	14.7 (13.4–16.1)^b^	30
1+ bacteria, no viruses	7.0 (6.5–7.4)	168	17.3 (15.9–18.6)	29
Mixed viral + bacterial	7.7 (7.2–8.2)	139	16.9 (15.2–18.6)	30
No virus or bacteria	6.8 (6.2–7.4)	100	18.4 (16.2–20.6)	30
Total	7.1 (6.9–7.3)	618	16.5 (15.7–17.3)	30

^a^ 90% percentile for duration in days.

^b^ ANOVA significant at F = 0.0096 for difference in duration between viral, bacterial, mixed, and no detection.

### 3.3 Univariate Analysis

The association between individual signs and symptoms and the type of infection is summarized in Table 3. Most associations for symptoms were statistically significant. With regard to signs and physician assessment, only the presence of diffuse wheeze on examination was significantly less likely with bacterial infections.

**TABLE 3:** Univariate analysis of signs and symptoms and their association with viral, bacterial, mixed infection, or no growth.

	Overall *n* = 618	Bacterial *n* = 168	Mixed *n* = 139	Viral *n* = 211	No detection *n* = 100	*p*
Patient reported symptom moderate or severe						
Cough	74.8%	77.4%	74.1%	71.1%	79.0%	0.376
Feeling generally unwell	73.3%	64.9%	81.3%	78.2%	66.0%	0.001
Fatigue	68.6%	62.5%	71.2%	74.4%	63.0%	0.044
Interference with activities	66.0%	61.9%	74.8%	69.7%	53.0%	0.002
Coryza	53.9%	42.3%	56.8%	67.8%	40.0%	< 0.001
Sputum	42.9%	47.6%	51.2%	32.9%	32.0%	0.009
Headache	41.4%	37.5%	38.1%	46.9%	41.0%	0.229
Myalgia	38.0%	28.6%	48.2%	42.6%	30.0%	0.001
Chest pain or discomfort	36.2%	27.4%	48.2%	35.1%	37.0%	0.002
Chest congestion	36.1%	29.2%	43.9%	37.9%	33.0%	0.049
Loss of appetite	30.9%	24.4%	40.3%	33.7%	23.0%	0.005
Sputum colored	30.1%	42.9%	29.5%	23.2%	24.0%	< 0.001
Fever	29.6%	18.5%	36.7%	38.4%	20.0%	< 0.001
Chills or sweats	29.0%	17.9%	38.1%	36.0%	20.0%	< 0.001
Wheeze	27.8%	25.6%	33.1%	25.1%	30.0%	0.342
Shortness of breath	22.8%	17.3%	28.1%	19.4%	32.0%	0.010
Confusion	7.3%	4.2%	5.8%	10.9%	7.0%	0.072
Diarrhea	4.9%	3.0%	5.8%	6.2%	4.0%	0.482
Specific symptoms present regardless of severity						
Felt warm or feverish on every day of illness	49.7%	39.9%	56.5%	55.0%	33.3%	< 0.001
Cough causing shortness of breath or lightheadedness	49.7%	47.0%	58.7%	44.0%	55.6%	0.030
Cough causing nausea or vomiting	43.1%	44.6%	52.2%	38.8%	38.4%	0.063
Double‐sickening	39.5%	49.4%	34.5%	33.2%	43.0%	0.006
Clinician examination						
Any abnormal lung finding	13.4%	9.5%	15.1%	14.2%	16.0%	0.359
Rhonchi	4.7%	3.8%	6.0%	4.4%	5.3%	0.829
Diffuse wheeze	4.2%	0.63%	5.9%	4.4%	7.5%	0.038
Crackles	4.1%	3.8%	3.7%	4.4%	4.3%	0.988
Expiratory wheeze	4.1%	2.5%	4.5%	4.4%	5.3%	0.693
Diminished vesicular breathing	2.9%	2.5%	2.2%	3.4%	3.2%	0.915

### 3.4 Multivariate Analysis

Results of 3 logistic regressions, with viral, bacterial, or mixed infection respectively as the dependent variable, are summarized in Table 4. Four symptoms were significantly more likely with viral infections and less common in bacterial: coryza, confusion, fever, and chest congestion. Three symptoms were significantly more likely with bacterial infections and less likely with viral infections: presence of sputum, sputum that is colored, and double‐sickening (initially feeling better but then worsening). With regards to mixed infections, sputum and confusion resembled the associations for bacterial infections, while that for double‐sickening resembled that for viral infections. The full logistic regression results with confidence intervals are shown in Appendix S2.

**TABLE 4:** Logistic regression of symptoms for dependent variables of viral, bacterial, or mixed infection.

Sign or symptom	Adjusted odds ratio (aOR)			Viral aOR/bacterial aOR
	Viral	Bacterial	Mixed	
**Coryza**	**2.56**	**0.52**		4.9
**Confusion**	**2.31**	**0.53**	0.52	4.4
**Fever**	**1.63**	**0.56**		2.9
**Chest congestion**	**1.39**	**0.72**		1.9
**Sputum**	**0.65**	**1.42**	1.45	0.5
**Double‐sickening**	**0.65**	**1.80**	0.71	0.4
**Sputum is colored**	**0.64**	**2.34**		0.3
Cough with lightheadedness or shortness of breath	0.75		1.35	
Short of breath	0.60			
Chest discomfort		0.63	1.56	
Felt unwell			1.69	
Fatigue			0.59	
Appetite loss			1.47	
Persistent fever since cough onset			1.47	

*Note:* Bold face indicates a symptom more associated with viral and less with bacterial detection or vice versa. The three full regression models with confidence intervals are shown in Appendix S2.

The only significant association between a physical exam finding and the type of pathogen was the presence of diffuse wheeze, which was associated with a lower likelihood of bacterial infection. When added to the model, the adjusted odds ratio for the association between diffuse wheeze and bacterial infection was 0.09, although the 95% confidence interval was wide (0.01–0.78) (Appendix S3).

### 3.5 Exploratory Risk Score Analysis

We assigned 1 point for each of the four viral symptoms and −1 for each of the bacterial symptoms to create a simple risk score, shown in Table 5. In the subgroup of patients with a confirmed viral or a confirmed bacterial detection, it identifies groups with a low (29%), moderate (56%), and high (79%) likelihood of having a viral pathogen detected. The likelihood of requiring a follow‐up visit for their LRTI was 10% in the group with a low likelihood of a bacterial pathogen, 12% if the risk was moderate, and 19% if the risk of a bacterial was high. The likelihood of a follow‐up visit was 22% if the score was −2 or −3 (suggesting bacterial infection) and only 7.5% if the score was +2 or +3 (suggesting viral infection).

**TABLE 5:** Proposed simple risk score for distinguishing viral from bacterial infection.

Points	Bacterial detection	Viral detection	Total	% bacterial	% viral
−3	4	1	5	80.0%	20.0%
−2	25	4	29	86.2%	13.8%
−1	59	30	89	66.3%	33.7%
0	49	62	111	44.1%	55.9%
1	19	72	91	20.9%	79.1%
2	11	31	42	26.2%	73.8%
3	1	11	12	8.3%	91.7%
4	0	0	0		
	168	211	379		

Points	Bacterial detection	Viral detection	Total	% bacterial	% viral
−3 to −1	88	35	123	71.5%	28.5%
0	49	62	111	44.1%	55.9%
+1 to +4	31	114	145	21.4%	78.6%

*Note:* To calculate the score, +1 point is given for each viral symptom reported as moderate or severe (coryza, confusion, fever, chest congestion) and −1 point is given for each bacterial symptom reported by patients as moderate or severe (sputum present, sputum is colored, and double‐sickening).

## 4 Discussion

In this large study of adults presenting with a LRTI to primary or urgent care settings, we identified seven symptoms that are significantly associated with viral versus bacterial infection. Coryza, confusion, fever, and chest congestion were associated with detection of a viral pathogen by PCR, while the presence of sputum, sputum that is colored, and double‐sickening were associated with detection of bacterial pathogens.

While most of the associations have reasonable face validity, we were surprised by the strength of association between confusion and viral infection. The question specifically asked patients at the initial visit whether “Confusion or disorientation” was present, and if present whether it was mild, moderate, or severe. It was the least commonly reported symptom among the seven identified by our analysis, present in only 4.2% with bacterial infection, 5.8% with mixed infection, and 10.9% with viral infection.

All of these symptoms were patient‐reported and should be amenable for use during a telehealth consultation. These symptoms could be the basis for triage decisions regarding whether a patient is likely to have a viral infection. We developed a simple risk score to identify patients with low, moderate, and high likelihoods of viral or bacterial infection, although it requires prospective validation.

While some viral infections such as influenza and SARS‐CoV‐2 do have specific treatments and could benefit from early in‐person evaluation, they have limitations. Oseltamivir and baloxavir only reduce the duration of symptoms by about a day, do not reduce the likelihood of hospitalization, and have significant side effects [9, 10]. While nirmatrelvir/ritonavir is still recommended for COVID‐19 in older patients and those with comorbidities, it is of little value in otherwise healthy patients, especially if vaccinated. In the current omicron era with widespread vaccination and/or previous infections with COVID‐19, the hospitalization rate is approximately 1.2% [11]. Given a relative risk of 0.52 for hospitalization based on recent studies [12], the overall number needed to treat (NNT) to prevent one hospitalization with nirmatrelvir/ritonavir is approximately 200, which we judge to be of modest benefit.

Some high‐risk patients may benefit more from treatment. The validated 3‐item Lehigh Outpatient COVID Hospitalization (LOCH) risk score identifies outpatients in the Omicron era with a low (0.09%), moderate (0.91%), and high (4.9%) risk of hospitalization. Thus, treatment is of almost no value for low‐risk patients (40% of the overall population, NNT = 1111), is of modest benefit for moderate‐risk patients (also 40% of the population, NNT = 110), but is of clinically significant value in those at high risk (NNT = 21). The risk score does not require an in‐person evaluation, so high‐risk patients could be identified during triage. An important advantage of reducing patient exposure of average‐risk patients likely to have a viral infection to clinicians is that it greatly reduces or even eliminates the likelihood of an unnecessary antibiotic.

Being able to classify patients as likely viral infection (or likely bacterial) is especially relevant as we move into an era where home testing becomes more widely available. In the United States, home testing for influenza, respiratory syncytial virus, COVID‐19, and Group A streptococcus are already in wide use. Early results of home testing from the Seattle Flu Study suggest that this approach is feasible and may reduce the need for in‐person visits [1]. Additional studies are needed to understand how risk scores and home testing can be best utilized to optimize patient outcomes, identifying patients who can be safely managed at home and others who may benefit from a telehealth or in‐person consultation with a primary care physician.

### 4.1 Strengths and Limitations

Our study had several notable strengths. This was a relatively diverse patient population from 3 different geographic regions over 3.5 years. The PCR testing was performed by one of the top laboratories in the world at the CDC, and patients were typical of the US outpatient population. Limitations include the sample size, which, while substantial, still resulted in broad confidence intervals for some associations. While we propose a risk score, it requires external validation. Also, not all bacterial detections necessarily represent a pathogen and may simply be commensal. A separate analysis is addressing this question.

### 4.2 Summary

We identified four symptoms associated with detection of a virus (coryza, confusion, fever, and chest contusion) and three symptoms (sputum, sputum that is colored, and double‐sickening) that were associated with detection of a potential bacterial pathogen. Nearly 80% of patients with more viral than bacterial symptoms had a viral infection, and we propose that in most cases they could be managed safely at home without a physician visit and the possibility of an unneeded antibiotic prescription. Prospective validation of this risk score is needed.

## Author Contributions

**Mark Ebell:** conceptualization, funding acquisition, writing – original draft, methodology, formal analysis, project administration, data curation, writing – review and editing, supervision. **Dan J. Merenstein:** writing – review and editing, funding acquisition, conceptualization, supervision. **Bruce Barrett:** funding acquisition, conceptualization, writing – review and editing, supervision. **Cassie Hulme:** data curation, investigation, writing – review and editing. **Sarah Walters:** data curation, investigation, writing – review and editing. **Caroline Hamer:** data curation, investigation, writing – review and editing. **Michelle Buhr:** data curation, investigation, writing – review and editing.

## Funding

This study was supported by AHRQ grant #1R01HS025584‐01A1.

## Conflicts of Interest

The authors declare no conflicts of interest.

## Supporting information


**Appendix S1:** Viral and bacterial pathogens tested for in each patient.
**Appendix S2:** Detailed regression models.
**Appendix S3:** Logistic regression model for symptoms significant at p < 0.1 in the univariate analysis, adding diffuse wheeze as a physical exam finding. Results shown for both viral and bacterial infection as the dependent variable, only showing odds ratios that were statistically significant in each model.

## Data Availability

Data are available upon reasonable request for collaborative analysis from the authors.

## References

[1] X. Cai , M. H. Ebell , R. E. Geyer , M. Thompson , N. L. Gentile , and B. Lutz , “The Impact of a Rapid Home Test on Telehealth Decision‐Making for Influenza: A Clinical Vignette Study,” BMC Primary Care 23, no. 1 (2022): 75, 10.1186/s12875-022-01675-1. 35418027 PMC9006488

[2] J. Teepe , B. D. L. Broekhuizen , K. Loens , et al., “Predicting the Presence of Bacterial Pathogens in the Airways of Primary Care Patients With Acute Cough,” CMAJ 189, no. 2 (2017): E50–E55, 10.1503/cmaj.151364. 27777252 PMC5235925

[3] A. W. Graffelman , A. Knuistingh Neven , S. le Cessie , A. C. M. Kroes , M. P. Springer , and P. J. van den Broek , “A Diagnostic Rule for the Aetiology of Lower Respiratory Tract Infections as Guidance for Antimicrobial Treatment,” British Journal of General Practice 54, no. 498 (2004): 20–24. PMC1314773 14965402

[4] J. Macfarlane , W. Holmes , P. Gard , et al., “Prospective Study of the Incidence, Aetiology and Outcome of Adult Lower Respiratory Tract Illness in the Community,” Thorax 56, no. 2 (2001): 109–114. 11209098 10.1136/thorax.56.2.109 PMC1746009

[5] R. M. Hopstaken , E. E. Stobberingh , J. A. Knottnerus , et al., “Clinical Items Not Helpful in Differentiating Viral From Bacterial Lower Respiratory Tract Infections in General Practice,” Journal of Clinical Epidemiology 58, no. 2 (2005): 175–183, 10.1016/j.jclinepi.2004.08.004. 15680752

[6] M. H. Ebell , D. J. Merenstein , B. Barrett , et al., “Acute Cough in Outpatients: What Causes It, How Long Does It Last, and How Severe Is It for Different Viruses and Bacteria?,” Clinical Microbiology and Infection 30 (2024): S1198‐743X(24)00312–4, 10.1016/j.cmi.2024.06.031. 38977076

[7] S. Lehrl , H. Matthys , W. Kamin , and P. Kardos , “The BSS—A Valid Clinical Instrument to Measure the Severity of Acute Bronchitis,” Journal of Lung, Pulmonary & Respiratory Research 1, no. 3 (2014): 00016, 10.15406/jlprr.2014.01.00016.

[8] B. Krismer , C. Weidenmaier , A. Zipperer , and A. Peschel , “The Commensal Lifestyle of *Staphylococcus aureus* and Its Interactions With the Nasal Microbiota,” Nature Reviews. Microbiology 15, no. 11 (2017): 675–687, 10.1038/nrmicro.2017.104. 29021598

[9] M. H. Ebell , M. Call , and J. Shinholser , “Effectiveness of Oseltamivir in Adults: A Meta‐Analysis of Published and Unpublished Clinical Trials,” Family Practice 30, no. 2 (2013): 125–133, 10.1093/fampra/cms059. 22997224

[10] T. Jefferson , M. Jones , P. Doshi , and C. Del Mar , “Neuraminidase Inhibitors for Preventing and Treating Influenza in Healthy Adults: Systematic Review and Meta‐Analysis,” BMJ (Clinical Research Ed.) 339 (2009): b5106, 10.1136/bmj.b5106. PMC2790574 19995812

[11] M. H. Ebell , R. Hamadani , and A. Kieber‐Emmons , “Development and Validation of Simple Risk Scores to Predict Hospitalization in Outpatients with COVID‐19 Including the Omicron Variant,” Journal of the American Board of Family Medicine 38, no. 6 (2022): jabfm.2022.AP.220056, 10.3122/jabfm.2022.AP.220056. 36564190

[12] S. Ombelet , D. Castanares‐Zapatero , F. Desimpel , F. Hulstaert , S. Stordeur , and D. Roberfroid , “Effectiveness of Nirmatrelvir‐Ritonavir on Severe Outcomes of COVID‐19 in the Era of Vaccination and Omicron: An Updated Meta‐Analysis,” Journal of Medical Virology 96, no. 2 (2024): e29434, 10.1002/jmv.29434. 38376947

