# Supplementary material for: Symptoms Associated With Detection of Viral Versus Bacterial Pathogens in Outpatients With Lower Respiratory Infections
Source: Influenza Other Respir Viruses. 2026 Mar 15;20(3):e70229. doi: 10.1111/irv.70229 (PMC13097432; doi:10.1111/irv.70229)
Supplement: Supplementary file 1 — Appendix S1: Viral and bacterial pathogens tested for in each patient. Appendix S2: Detailed regression models. Appendix S3: Logistic regression model for symptoms significant at p < 0.1 in the univariate analysis, adding diffuse wheeze as a physical exam finding. Results shown for both viral and bacterial infection as the dependent variable, only showing odds ratios that were statistically significant in each model. [file IRV-20-e70229-s001.docx]

**Appendix A. Viral and bacterial pathogens tested for in each patient**

| **Organism** | **Organism** |
| --- | --- |
| Adenovirus | Acinetobacter baumanii |
| Cytomegalovirus | Bordetella (IS481) |
| Enterovirus | Bordetella pertussis toxin |
| H. influenzae (all types) | Bordetella (IS1001) |
| H. influenzae type B | Burkholderia pseudomallei |
| Human coronavirus 1 | Chlamydia pneumoniae |
| Human coronavirus 2 | Chlamydia trachomatis |
| Human coronavirus 3 | C. diphtheriae |
| Human coronavirus 4 | C. ulcerans/pseudotuberculosis |
| Human metapneumovirus | Corynebacterium tox gene |
| Influenza A | Group A Strep (S. pyogenes) |
| Influenza B | Group B strep |
| Measles virus | Klebsiella pneumoniae |
| MERS N | Moraxella catarrhalis |
| MERS upE | Mycoplasma pneumoniae |
| Parainfluenza virus type 1 | Mycobacterium tuberculosis |
| Parainfluenza virus type 2 | Pneumocysitis jirovecii |
| Parainfluenza virus type 3 | P. aeruginosa |
| Parainfluenza virus type 4 | S. aureus |
| Rubella | Streptococcus pneumoniae |
| Respiratory synytical virus | Ureaplasma parvum |
| Rhinovirus | Ureaplasma urealyticum |
| SARS-CoV-2 | Varicella zoster |

**Appendix B. Detailed regression models**

. *** LR 0: Only symptoms where p < 0.1 in univariate, pr(0.2), stepwise backward eliimination

**Dependent variable = viral infection**

. stepwise, pr(0.2): logistic num_infection_viral fever_modsevere unwell_modsevere fatigue_modse

> vere coryza_modsevere myalgia_modsevere chestcongest_modsevere chestpain_modsevere appetitelos

> s_modsevere chillsorsweats_modsevere sob_modsevere confusion_modsevere sputum_modsevere interf

> ereactivity_modsevere usualactive_modsevere double_sickening sputum_colored nausea_with_cough_

> num sob_lightheaded_cough_num persistentfever_cough_num if symptomatic==1 & validspecimen==1

note: usualactive_modsevere omitted because of estimability.

Wald test, begin with full model:

p = 0.9080 >= 0.2000, removing unwell_modsevere

p = 0.9062 >= 0.2000, removing myalgia_modsevere

p = 0.7930 >= 0.2000, removing chillsorsweats_modsevere

p = 0.7703 >= 0.2000, removing appetiteloss_modsevere

p = 0.7816 >= 0.2000, removing interfereactivity_modsevere

p = 0.6840 >= 0.2000, removing chestpain_modsevere

p = 0.3890 >= 0.2000, removing nausea_with_cough_num

p = 0.3410 >= 0.2000, removing persistentfever_cough_num

p = 0.2866 >= 0.2000, removing fatigue_modsevere

Logistic regression Number of obs = 618

LR chi2(9) = 64.26

Prob > chi2 = 0.0000

Log likelihood = -364.6089 Pseudo R2 = 0.0810

-------------------------------------------------------------------------------------------

num_infection_viral | Odds ratio Std. err. z P>|z| [95% conf. interval]

--------------------------+----------------------------------------------------------------

fever_modsevere | 1.625639 .3234884 2.44 0.015 1.100633 2.401077

confusion_modsevere | 2.310574 .7860715 2.46 0.014 1.18614 4.500947

sob_modsevere | .604338 .154501 -1.97 0.049 .3661571 .9974527

coryza_modsevere | 2.56199 .4964827 4.85 0.000 1.752366 3.745674

sob_lightheaded_cough_num | .7477612 .1484083 -1.46 0.143 .5067862 1.103319

chestcongest_modsevere | 1.387164 .2993991 1.52 0.129 .9086742 2.117616

double_sickening | .6498317 .1235722 -2.27 0.023 .4476475 .9433344

sputum_colored | .6382967 .137492 -2.08 0.037 .4184754 .973588

sputum_modsevere | .6514142 .133191 -2.10 0.036 .4363317 .972518

_cons | .442303 .078584 -4.59 0.000 .3122388 .626546

-------------------------------------------------------------------------------------------

Note: _cons estimates baseline odds.

**Dependent variable = bacterial infection**

. stepwise, pr(0.2): logistic num_infection_bacterial fever_modsevere unwell_modsevere fatigue_m

> odsevere coryza_modsevere myalgia_modsevere chestcongest_modsevere chestpain_modsevere appetit

> eloss_modsevere chillsorsweats_modsevere sob_modsevere confusion_modsevere sputum_modsevere in

> terfereactivity_modsevere usualactive_modsevere double_sickening sputum_colored nausea_with_co

> ugh_num sob_lightheaded_cough_num persistentfever_cough_num if symptomatic==1 & validspecimen=

> =1

note: usualactive_modsevere omitted because of estimability.

Wald test, begin with full model:

p = 0.7089 >= 0.2000, removing persistentfever_cough_num

p = 0.6493 >= 0.2000, removing fatigue_modsevere

p = 0.6489 >= 0.2000, removing myalgia_modsevere

p = 0.6343 >= 0.2000, removing sob_modsevere

p = 0.5403 >= 0.2000, removing sob_lightheaded_cough_num

p = 0.4792 >= 0.2000, removing appetiteloss_modsevere

p = 0.4989 >= 0.2000, removing nausea_with_cough_num

p = 0.4279 >= 0.2000, removing unwell_modsevere

p = 0.3865 >= 0.2000, removing interfereactivity_modsevere

p = 0.2642 >= 0.2000, removing chillsorsweats_modsevere

Logistic regression Number of obs = 618

LR chi2(8) = 65.16

Prob > chi2 = 0.0000

Log likelihood = -329.00361 Pseudo R2 = 0.0901

-----------------------------------------------------------------------------------------

num_infection_bacterial | Odds ratio Std. err. z P>|z| [95% conf. interval]

------------------------+----------------------------------------------------------------

fever_modsevere | .5562411 .132442 -2.46 0.014 .3488112 .8870247

confusion_modsevere | .5344507 .2410325 -1.39 0.165 .2208123 1.293576

double_sickening | 1.80504 .3548752 3.00 0.003 1.227831 2.653598

coryza_modsevere | .5220006 .1065438 -3.19 0.001 .3498929 .778766

sputum_modsevere | 1.42224 .3079346 1.63 0.104 .9304132 2.174051

chestcongest_modsevere | .719629 .1760465 -1.34 0.179 .445528 1.162365

chestpain_modsevere | .626284 .1483393 -1.98 0.048 .3936941 .9962852

sputum_colored | 2.344064 .5006459 3.99 0.000 1.542302 3.562619

_cons | .4081966 .0715008 -5.12 0.000 .2895814 .5753978

-----------------------------------------------------------------------------------------

Note: _cons estimates baseline odds.

**Dependent variable = mixed infection**

. stepwise, pr(0.2): logistic num_infection_mixed fever_modsevere unwell_modsevere fatigue_modse

> vere coryza_modsevere myalgia_modsevere chestcongest_modsevere chestpain_modsevere appetitelos

> s_modsevere chillsorsweats_modsevere sob_modsevere confusion_modsevere sputum_modsevere interf

> ereactivity_modsevere usualactive_modsevere double_sickening sputum_colored nausea_with_cough_

> num sob_lightheaded_cough_num persistentfever_cough_num if symptomatic==1 & validspecimen==1

note: usualactive_modsevere omitted because of estimability.

Wald test, begin with full model:

p = 0.9841 >= 0.2000, removing chestcongest_modsevere

p = 0.9382 >= 0.2000, removing coryza_modsevere

p = 0.9210 >= 0.2000, removing sob_modsevere

p = 0.6038 >= 0.2000, removing interfereactivity_modsevere

p = 0.5333 >= 0.2000, removing fever_modsevere

p = 0.3834 >= 0.2000, removing myalgia_modsevere

p = 0.3033 >= 0.2000, removing sputum_colored

p = 0.3057 >= 0.2000, removing nausea_with_cough_num

p = 0.2201 >= 0.2000, removing chillsorsweats_modsevere

Logistic regression Number of obs = 618

LR chi2(9) = 31.95

Prob > chi2 = 0.0002

Log likelihood = -313.45695 Pseudo R2 = 0.0485

-------------------------------------------------------------------------------------------

num_infection_mixed | Odds ratio Std. err. z P>|z| [95% conf. interval]

--------------------------+----------------------------------------------------------------

double_sickening | .7133283 .1489568 -1.62 0.106 .4737422 1.074081

unwell_modsevere | 1.687648 .5574645 1.58 0.113 .8833093 3.224416

fatigue_modsevere | .5946926 .1752232 -1.76 0.078 .333803 1.059485

sob_lightheaded_cough_num | 1.346043 .2876447 1.39 0.164 .885442 2.046245

sputum_modsevere | 1.446188 .2943471 1.81 0.070 .9704586 2.155125

persistentfever_cough_num | 1.468407 .3009155 1.87 0.061 .9826811 2.19422

chestpain_modsevere | 1.56183 .3363381 2.07 0.038 1.024068 2.381985

appetiteloss_modsevere | 1.473389 .3296332 1.73 0.083 .9503481 2.284294

confusion_modsevere | .5229258 .2196048 -1.54 0.123 .2296013 1.190983

_cons | .1445737 .0371234 -7.53 0.000 .0874016 .2391439

-------------------------------------------------------------------------------------------

Note: _cons estimates baseline odds.

**Appendix C. Logistic regression model for symptoms significant at p < 0.1 in the univariate analysis, adding diffuse wheeze as a physical exam finding. Results shown for both viral and bacterial infection as the dependent variable, only showing odds ratios that were statistically significant in the each model.**

|  | **Adjusted odds ratio (95% CI)** | |
| --- | --- | --- |
| **Sign or symptom** | **Viral infection** | **Bacterial infection** |
| Fever | 1.65 (1.10-2.40) | 0.67 (0.38-1.21) |
| Double-sickening | 0.65 (0.45-0.94) | 1.73 (1.18-2.53) |
| Short of breath | 0.60 (0.37-0.99) |  |
| Coryza | 2.56 (1.75-3.74) | 0.50 (0.34-0.75) |
| Sputum is colored | 0.64 (0.42-0.97) | 2.30 (1.51-3.51) |
| Chest congestion | 1.39 (0.91-2.12) |  |
| Confusion | 2.31 (1.18-4.50) |  |
| Cough with lightheadedness and shortness of breath | 0.75 (0.51-1.10) |  |
| Sputum | 0.65 (0.44-0.97) | 1.37 (0.90-2.09) |
| Diffuse wheeze |  | 0.10 (0.01-0.78) |
| Chills or sweats |  | 0.66 (0.36-1.19) |
| Chest discomfort |  | 0.57 (0.37-0.87) |
